# The global Edoxaban Treatment in routine cliNical prActice (ETNA) noninterventional study program: rationale and design

**DOI:** 10.1002/clc.23279

**Published:** 2019-10-25

**Authors:** Raffaele De Caterina, Giancarlo Agnelli, Petra Laeis, Martin Unverdorben, Heiko Rauer, Chun‐Chieh Wang, Mashio Nakamura, Kuan‐Ming Chiu, Paul‐Egbert Reimitz, Yukihiro Koretsune, Cathy Chen, Ulrike Thee, Jumpei Kaburagi, Young‐Hoon Kim, Won‐Il Choi, Takeshi Yamashita, Alexander Cohen, Paulus Kirchhof

**Affiliations:** ^1^ Chair of Cardiology University of Pisa Pisa Italy; ^2^ Internal and Cardiovascular Medicine‐Stroke Unit University of Perugia Perugia Italy; ^3^ Clinical Operations and Biostatistics and Data Operations Daiichi Sankyo Europe GmbH Munich Germany; ^4^ Global Medical Affairs Daiichi Sankyo, Inc. Basking Ridge New Jersey; ^5^ Cardiology, Chang Gung Memorial Hospital Chang Gung University Taoyuan Taiwan Republic of China; ^6^ Department of Internal Medicine Pediatrics and Cardiology, Nakamura Medical Clinic Kuwana Mie Japan; ^7^ Cardiovascular Center Far Eastern Memorial Hospital New Taipei City Taiwan Republic of China; ^8^ Electrical Engineering Yuan Ze University Taoyuan City Taiwan Republic of China; ^9^ National Hospital Organization Osaka National Hospital Osaka Japan; ^10^ Medical Science Department Daiichi Sankyo Tokyo Japan; ^11^ Department of Internal Medicine Korea University College of Medicine and Korea University Medical Center Seoul Republic of Korea; ^12^ Department of Internal Medicine Keimyung University Dongsan Hospital Daegu Republic of Korea; ^13^ Department of Cardiovascular Medicine Cardiovascular Institute Tokyo Japan; ^14^ Guy's and St Thomas' NHS Foundation Trust King's College London London UK; ^15^ Institute of Cardiovascular Sciences University of Birmingham and SWBH and UHB NHS Trusts of Birmingham, School of Clinical and Experimental Medicine Birmingham UK

## Abstract

**Background:**

Randomized controlled trials showed the nonvitamin K oral anticoagulant (NOAC) edoxaban was effective and safe for stroke and systemic embolism prevention in nonvalvular atrial fibrillation (AF) and for the prevention and treatment of venous thromboembolism (VTE; including pulmonary embolism and deep vein thrombosis). Additional research is needed to evaluate the effects of edoxaban in routine clinical practice. Therefore, the Edoxaban Treatment in routine cliNical prActice (ETNA) program is being conducted to provide routine clinical care data on characteristics and outcomes in patients with AF or VTE receiving edoxaban.

**Methods:**

The Global ETNA program integrates prospectively collected data from edoxaban patients in regional ETNA noninterventional studies across Europe, Japan, and East and Southeast Asia into indication‐specific databases for AF and VTE. Targeted enrollment is >31 000 patients (AF >26 000; VTE >4500), with a follow‐up of 2 years for AF and 1 year for VTE. Data integration will be possible using consistent terminology, parameter definitions, and data collection across the regional noninterventional studies. Safety and effectiveness data will be assessed. Crude rates of outcomes including bleeding and thromboembolic events will be reported.

**Results:**

Globally, enrollment began in early 2015 and is ongoing.

**Conclusions:**

Global ETNA will generate the largest integrated prospective repository of routine clinical care data for a single NOAC in patients with AF or VTE. It will provide important information on the safety of edoxaban in routine clinical care and gather further information on its effectiveness.

## 1 INTRODUCTION

Vitamin K antagonist (VKA) oral anticoagulants such as warfarin have demonstrated efficacy for stroke prevention in patients with nonvalvular atrial fibrillation (AF) and prevention and treatment of venous thromboembolism (VTE).^1^, ^2^ The non‐VKA oral anticoagulant (NOAC) edoxaban directly inhibits factor Xa to prevent thromboembolism.^3^ In the Effective aNticoaGulation with Factor Xa next GEneration in Atrial Fibrillation‐Thrombolysis in Myocardial Infarction study 48 (ENGAGE AF‐TIMI 48), once‐daily edoxaban exhibited similar efficacy for the prevention of stroke or systemic embolic events (SEE) and significantly lower rates of bleeding and death from cardiovascular causes compared with well‐managed warfarin in patients with AF.^3^ These results are broadly comparable to the other NOACs—dabigatran, rivaroxaban, and apixaban.^4^ In the Hokusai‐VTE study, once‐daily edoxaban following initial parenteral anticoagulant therapy was noninferior to warfarin for the treatment and prevention of recurrent VTE and associated with significantly fewer major and clinically relevant nonmajor bleeding events.^5^ However, there is little data on the safety and effectiveness of edoxaban in routine clinical practice settings.

Therefore, regional Edoxaban Treatment in routine cliNical prActice (ETNA) noninterventional studies were independently implemented to collect data on patient characteristics and routine clinical care outcomes in patients treated with edoxaban for stroke prevention in AF and for treatment of VTE, with the intention to integrate data through consistent a priori definitions of patient characteristics and outcomes. This design will allow for the integration of all information into Global ETNA‐AF and ETNA‐VTE databases. The set‐up will also allow for combining the AF and VTE databases.

Here, we describe the design and methodology of the Global ETNA program, the common parameters (ie, the so‐called “core data”), the observational periods, the program's differentiation from other NOAC noninterventional studies, and other technical details such as the consistency of terminology.

## 2 METHODS

### 2.1 Design

The Global ETNA program encompasses several multinational, prospective, observational noninterventional studies, including a European noninterventional observational study (Germany, Austria, Switzerland, Belgium, Italy, Spain, UK, Ireland, the Netherlands, and Portugal)^6^, ^7^ and similar noninterventional observational studies in Japan^8^, ^9^ and other East and Southeast Asian countries that are intended to collect regular clinical care data in edoxaban patients with AF or acute or recurrent VTE (Supplemental Table 1). Within the Global ETNA‐AF and VTE program, regional ETNA‐AF and VTE studies are registered separately. Additional countries may be added as the study progresses. Altogether, the target global enrollment for these noninterventional studies is >31 000 edoxaban‐treated patients, >26 000 of whom will have AF and > 4500 will have VTE (Table 1).

**Table 1:** Target patient enrollment and number of sites for ETNA‐AF and ETNA‐VTE per country and/or region in noninterventional studies currently open or enrolling

	ETNA‐AF		ETNA‐VTE	
	Patients	Sites	Patients	Sites
Japan	10 000	1367	≥1500	281
Europe	13 100	825	2700	282
Other East and Southeast Asian countries	3500	63	500	20
Total number of patients	26 100		4550	

Abbreviations: AF, atrial fibrillation; ETNA, Edoxaban Treatment in routine cliNical prActice; VTE, venous thromboembolism.

Power analyses for sample size justification were performed on a regional basis. Detailed sample size justification for ETNA‐AF and ETNA‐VTE Europe has been published previously.^6^, ^7^ In ETNA‐AF Japan, a total of 10 000 patients will be enrolled. Based on the interim analysis of a postmarketing surveillance of another new orally active anticoagulant, 2% of patients enrolled are expected to have severe renal impairment. The annual rate of major bleeding in patients without severe renal impairment is assumed to about 2.5% based on the results of a global AF phase 3 study^3^; the risk in patients with severe renal impairment has more than doubled. Given these assumptions, at least 8500 patients are required to detect the risk in patients with severe renal impairment during 2 years follow‐up with 80% power and two‐sided α of 5%. Assuming an expected dropout rate of 15% after 2 years follow‐up, planned enrollment is set at 10000 patients. For ETNA‐VTE Japan, a total of 1500 patients will be enrolled. According to epidemiological data, an estimated 14 674 and 7864 Japanese patients are newly diagnosed with DVT and PE, respectively, per year.^10^ Given that about 60% of patients with PE also have DVT, about 18 000 Japanese patients will develop VTE per year. Together with the expected percentage of participating institutions, VTE patients treated with edoxaban, and the feasibility of patient accrual, the planned sample size was set at 1500 or more. Assuming the incidence of major or CRNM bleeding in patients treated with edoxaban for VTE is 8.5% based on the results of a global VTE phase 3 study,^5^ a sample size of 1500 will power this study to estimate incidence at 8.5% ± 1.4% (95% CI). Assuming the incidence of recurrent VTE in the same population is 3.2%, this study is powered to estimate the incidence of recurrent VTE at 3.2% ± 0.9% (95% CI).

The ETNA protocols were approved in all participating regions and countries by the responsible ethics committees (EC) and institutional review boards (IRB) prior to initiation, except Japan, where EC/IRB approval is not required for postmarketing surveillance programs. In Japan, ETNA‐AF (UMIN000017011) and ETNA‐VTE (UMIN000016387) are conducted as part of regulatory‐required postmarketing surveillance as drug use‐results surveys; protocols were reviewed by the Pharmaceuticals and Medical Devices Agency (PMDA) prior to initiation of the noninterventional study. ETNA‐AF Europe and ETNA‐VTE Europe were designed as postauthorization safety studies as part of the risk management plan of edoxaban in order to assess the risks and benefits of the drug in European patients. The design of ETNA‐AF and VTE Europe was agreed in close collaboration with the Pharmacovigilance Risk Assessment Committee (PRAC) of EMA as a single‐arm prospective international observational study. There have been no protocol amendments following enrollment of the first patients.

Potential sites where edoxaban has been used were identified using multiple databases and selected via a stepwise process to allow representative regional distribution of types of sites and specialties. All ETNA noninterventional study participants must provide written informed consent before enrollment. Consecutive enrollment is encouraged, but not mandated; sites are not recording an exclusion list. Patients will only be included in the study after the treating physician has made the clinical decision to prescribe edoxaban for AF or an acute VTE event. Only noninterventional observations derived from routine medical care will be collected. A patient with both AF and an acute VTE event can be enrolled in only one of ETNA‐AF and ETNA‐VTE. The conduct of this project will adhere to the Declaration of Helsinki and the International Council on Harmonization Good Clinical Practice guidelines.^11^, ^12^

### 2.2 Goals

The primary goal of the Global ETNA program is to integrate regional noninterventional study data into the Global databases to evaluate routine clinical usage, safety, and effectiveness of edoxaban. Individual regional ETNA noninterventional studies will obtain data on patients with AF or VTE who receive edoxaban treatment in routine clinical practice. Region‐specific findings will be reported separately. Data that are common to regional noninterventional studies will be integrated into indication‐specific global databases for analyses. This critical step allows analyses of substantially higher patient numbers than could be accomplished with individual regional studies.

### 2.3 Inclusion and exclusion criteria

For ETNA‐AF, eligible patients are those treated with edoxaban for AF according to the local label. Patients enrolled in ETNA‐AF Japan were only eligible if they were receiving edoxaban for the first time to prevent ischemic stroke and systemic embolism.^9^ Patients with atrial flutter only are not eligible for enrollment in ETNA‐AF.

For ETNA‐VTE, eligible patients are those diagnosed with initial or recurrent acute VTE and treated with edoxaban according to the local label. Patients enrolled in ETNA‐VTE Europe were only eligible if the diagnosis of initial or recurrent acute VTE occurred no more than 2 weeks prior to enrollment.^7^

Due to the noninterventional design, the only exclusion criteria are not providing written informed consent or participating in a simultaneous interventional study.

### 2.4 Common assessments and outcomes of the ETNA noninterventional studies

The common variables include the main outcome measures: the proportions of patients who experience clinically relevant events (Tables 2 and 3). As defined in the protocols, clinically relevant events include first or recurrent stroke; SEE; bleeding; transient ischemic attack; acute coronary syndrome; congestive heart failure; recurrent deep vein thrombosis or pulmonary embolism; and all‐cause, cardiovascular, or VTE‐related death. Bleeding events will be defined as major, clinically relevant nonmajor, or minor bleeding according to the criteria of the International Society on Thrombosis and Hemostasis.^13^ Further analyses will evaluate risk factors for death, stroke, and bleeding, and comparisons between patients receiving different doses according to regional labels.

**Table 2:** Outcomes for ETNA‐AF

Primary outcomes	Bleeding events including intracranial hemorrhage Drug‐related adverse events such as liver adverse events Cardiovascular and all‐cause mortality in AF patients
Secondary outcomes	Stroke (ischemic and hemorrhagic) Systemic embolic events Transient ischemic attack Major adverse cardiovascular events, a composite endpoint of nonfatal myocardial infarction, nonfatal stroke, nonfatal SEE, and death due to a cardiovascular cause or bleeding VTE episodes Acute coronary syndromes Hospitalizations related to cardiovascular condition Extent of exposure and compliance to edoxaban therapy, rate, and reasons of permanent discontinuation of edoxaban

Abbreviations: AF, atrial fibrillation; SEE, systemic embolic event; VTE, venous thromboembolism.

**Table 3:** Outcomes for ETNA‐VTE

Primary outcomes	Recurrent symptomatic VTE on one or more occasions
Secondary outcomes	Death (all‐cause, cardiovascular, and VTE‐related) Bleeding events (major, CRNM, and minor, defined by ISTH criteria Hepatic events Other adverse drug events Bleeding, ischemic/hemorrhagic stroke, SEE, post‐thrombotic syndrome, death, and hospitalization due to cardiovascular causes Reason for and rate of discontinuation

Abbreviations: CRNM, clinically relevant nonmajor bleeding; ISTH, International Society of Thrombosis and Hemostasis; SEE, systemic embolic event; VTE, venous thromboembolism.

**Figure 1:**
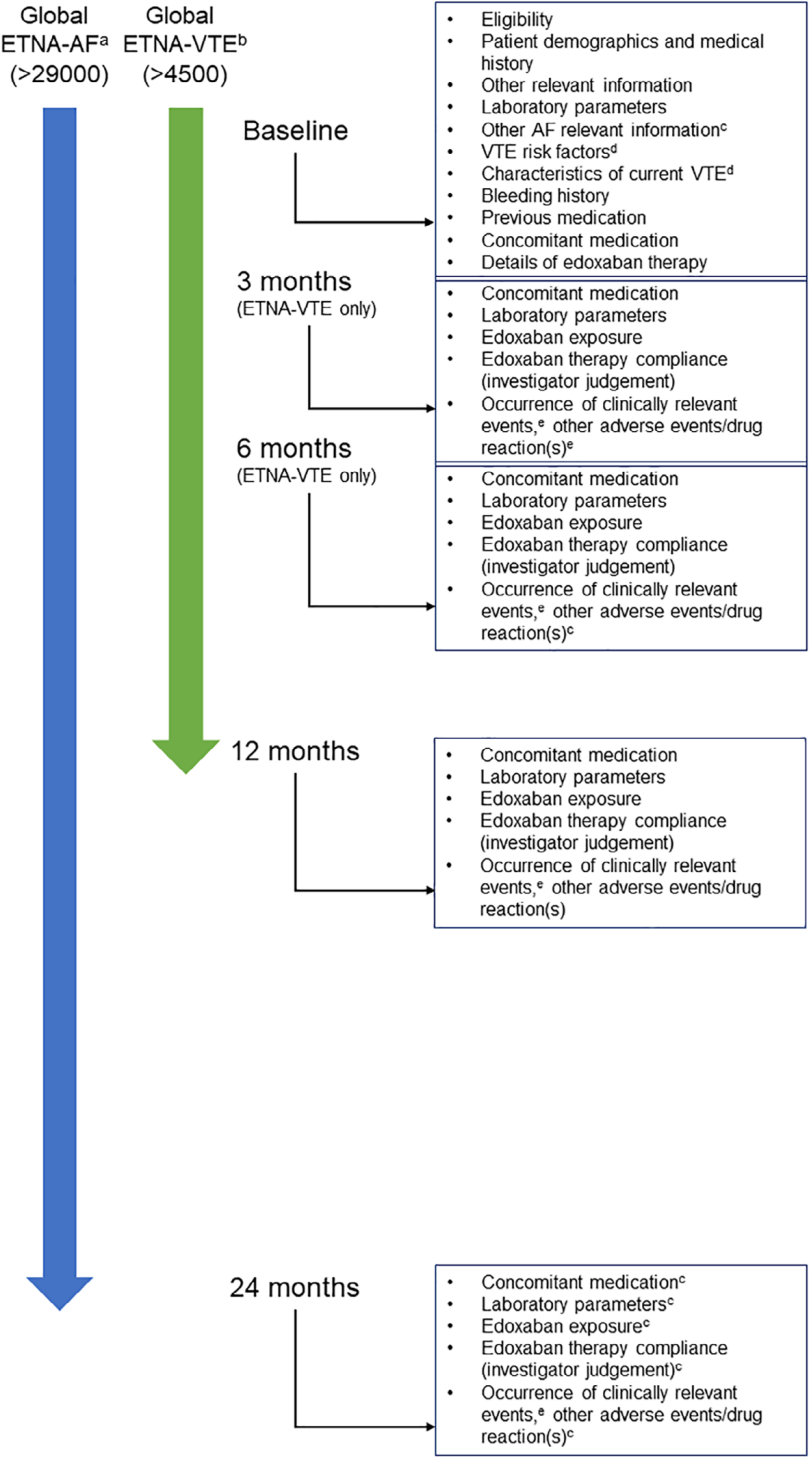
Timing of data documentation in the Global ETNA noninterventional studies. ^a^Follow‐up time for ETNA‐AF EU, Japan, and East and Southeast Asia is 4, 2, and 2 years, respectively; ^b^Follow‐up time for ETNA‐VTE EU, Japan, and East and Southeast Asia is 1.5, 1, and 1 years, respectively; ^c^ETNA‐AF only; ^d^ETNA‐VTE only; ^e^Investigator reported bleeding, stroke, SEE, TIA, ACS, HF, MI, VTE (and recurrent DVT and PE in ETNA‐VTE), and death. ACS, acute coronary syndrome; AF, atrial fibrillation; DVT, deep vein thrombosis; ETNA, Edoxaban Treatment in routine cliNical prActice; HF, heart failure; MI, myocardial infarction; PE, pulmonary embolism; SEE, systemic embolic event; TIA, transient ischemic attack; VTE, venous thromboembolism

Initiation and reasons for permanent discontinuation of edoxaban will be documented, as well as information on adherence to edoxaban treatment. Data on prior anticoagulant use, including reasons for switching anticoagulants, will be collected. The follow‐up period of the regional noninterventional studies ranges from 2 to 4 years for ETNA‐AF and from 1 to 1.5 years for ETNA‐VTE; durations of follow‐up are based on regulatory authority requirements in Japan and Europe (Figure 1). Therefore, in the Global ETNA program, the consolidated follow‐up periods are 2 years for all AF patients and 1 year for all VTE patients.

### 2.5 Documentation and data assessment

Data for the ETNA noninterventional studies will be prospectively derived from medical records and the treating physicians. For the Global ETNA‐AF program, data will be collected at baseline, 1 year, and 2 years after enrollment (Figure 1). In the Global ETNA‐VTE program, data will be collected at baseline and 3, 6, and 12 months (Figure 1). Patients who permanently discontinue edoxaban during the observation period will be followed‐up for 1 year beyond discontinuation or until the end of the trial observational period, whichever comes first.

Predefined variables and definitions will be used, and free text field documentation will be limited as much as possible. Electronic case report forms (eCRFs) will be utilized in all countries and noninterventional studies except for ETNA‐VTE Japan, where paper case report forms will be completed and transcribed via double data entry into eCRFs.

### 2.6 Data management and integration

Observational plans, CRFs and other documentation, and databases have been implemented independently in the three regions, reflecting regional requirements and differences in research governance and health care provision (Tables 2 and 3). Standardization and harmonization of data management processes based on regional and local ETNA data management processes and structures will be implemented to provide similar data quality, focusing on a unified core data set unless otherwise required by local circumstances. Data will be regularly processed and transferred into the global integrated databases throughout the study period to enable timely data analyses.

The global integrated databases will contain all core data from the regional and country‐specific ETNA noninterventional studies (Tables 2 and 3). Medical history, clinical events and descriptions, and adverse events/drug reactions will be coded using the Medical Dictionary for Regulatory Activities (MedDRA). Clinical events are based on physician diagnosis and assessment. Medications/drugs will be coded using the World Health Organization Drug Dictionary Enhanced (WHO‐DDE). If regional coding is required using regional/local dictionaries, a conversion into the globally required dictionaries will be performed when needed (eg, Japanese Iyakuhinmei Data File into WHO‐DDE; J‐MedDRA into E‐MedDRA) using standard tools from MedDRA Maintenance and Support Services Organization and/or the Uppsala Monitoring Centre.

### 2.7 Quality control

All ETNA noninterventional studies will be conducted in accordance with national laws and regulations. This includes Item 4 of Article 14‐4 of the Pharmaceutical Affairs Law (regarding drug re‐examination) and Good Post‐Marketing Surveillance Practice in Japan. The Guidelines for Good Pharmacoepidemiology Practice and the European Medicines Agency (EMA) Guideline on Good Pharmacovigilance Practices Module VIII (management and reporting of adverse reactions to medicinal products; EMA/813938/2011 rev 1) will be adhered to in Europe, Korea, and other East Asian countries. ETNA‐AF Europe and ETNA‐VTE Europe form part of the postapproval safety plan mandated by EMA, and the protocols have been reviewed and approved by EMA. ETNA‐AF and ETNA‐VTE Japan are conducted as postmarking surveillance studies, and protocols and eCRFs were reviewed by PMDA.

The regional and country‐specific ETNA noninterventional study teams are accountable for data collection, cleaning, and validation. Additional validation and quality control will be performed globally after each data integration step, allowing feedback with regional teams during data collection. These quality control activities will ensure that the reported data are accurate and complete as possible in a routine clinical care setting and that the conduct of all ETNA noninterventional studies is compliant with the ETNA observational plans and all applicable regional/local regulatory requirements.

### 2.8 Statistical analyses

All ETNA program data analyses will be exploratory and descriptive. Most of the analyses will be performed separately for Global ETNA‐AF and Global ETNA‐VTE. In general, all results will be presented by appropriate summary statistics overall and separately by edoxaban dose (30 or 60 mg). In addition, the results may be further broken down by other subgroups (eg, geographic region, country, gender, age, specific medical history, on‐ or off‐label use, etc). Two‐sided confidence intervals will be presented.

Baseline data will be presented as frequencies or with appropriate summary statistics (n, mean, SD, minimum, lower quartile, median, upper quartile, maximum). Patient characteristics in Global ETNA‐AF and Global ETNA‐VTE will be compared with those of the participants in ENGAGE AF‐TIMI 48 and the Hokusai‐VTE trials, respectively. The primary focus of follow‐up data analyses will be the incidence of clinically relevant events and adverse drug reactions. The number of subjects with at least one event will be presented (n [%]) separately for each type of clinical event. Crude event rates will be presented, and Kaplan‐Meier estimates of event risk will also be considered. Adverse drug reactions will be summarized by System Organ Class and Preferred Term based on MedDRA. Risk factors for stroke, bleeding, and discontinuation of therapy will be evaluated.

Data snapshot analyses for baseline and follow‐up data are planned at 1 and 2 years for AF and 6 and 12 months for VTE globally, followed by final analyses of baseline and complete follow‐up data. Secular trends in outcomes and patient characteristics will also be analyzed. In addition to separate reports on the Global ETNA‐AF and ‐VTE data sets, a combined analysis may be performed. Planned clinically relevant subpopulation analyses include by age, history of intracranial hemorrhage, recommended dose vs nonrecommended dose, and concomitant therapy.

## 3 RESULTS

Globally, enrollment began in early 2015 and is still ongoing. The number of participating sites per region can be seen in Table 1.

## 4 DISCUSSION

By combining and integrating all regional and country‐specific noninterventional study data at a global level, the Global ETNA program is the largest integrated prospective observational program of a single NOAC, collecting detailed information on the use, safety, and effectiveness of edoxaban from more than 31 000 patients with AF or VTE and approximately 50 000 patient‐years of exposure to edoxaban. The planned enrollment of 26 000 edoxaban patients in the Global ETNA‐AF program will exceed the combined number of patients receiving a NOAC at diagnosis included in the large prospective global AF noninterventional studies GARFIELD‐AF, GLORIA‐AF, and EORP‐AF^14^, ^15^, ^16^, ^17^, ^18^; the rivaroxaban for AF routine clinical practice observational study program XANTUS has also reported lower patient numbers.^19^ As such, Global ETNA can generate a large dataset for detailed analyses. In addition, ETNA‐AF includes all‐comers with AF treated with edoxaban, while GARFIELD‐AF, EORP‐AF, and GLORIA‐AF only focus on patients newly diagnosed with AF.^18^, ^20^, ^21^

A key strength of the ETNA program is that the regional noninterventional studies are designed specifically to meet region‐specific postmarketing requirements with mandated quality standards.

The Global ETNA program will continue to include more countries and regular snapshot analyses are planned. Similar to other large prospective global AF data sets (eg, >2 years for GARFIELD‐AF; 2‐3 years for GLORIA‐AF; 3 years for EORP‐AF; and 1 year for XANTUS), the combined follow‐up period for the Global program will be 2 years for ETNA‐AF and 1 year for ETNA‐VTE (Figure 1).^17^, ^18^, ^19^, ^21^

The integrated design of the Global ETNA core data addresses key questions about the clinical usage of edoxaban (Tables 2 and 3). These include whether the baseline clinical characteristics and outcomes in routine clinical usage of edoxaban reflect those of the pivotal randomized controlled trials and whether potential differences of dosing in routine clinical care compared with randomized clinical trials impact safety and effectiveness. Questions regarding the existence of regional usage patterns, how they affect outcomes, and how patient characteristics change over time following the introduction of edoxaban in different regions of the world can also be addressed. Finally, the timing of clinical events relative to the start of dosing, dosing interruption, resumption of dosing, or any situation leading to dose interruption can be examined.

Data on practical considerations for the use of edoxaban will be collected. For patients with AF, these include use of edoxaban with concomitant antiplatelet agents, potential differences depending on the type of antiplatelet agents (ie, aspirin, P_2_Y_12_ inhibitors), and the effects of concomitant single or dual antiplatelet therapy. The safety and effectiveness of edoxaban in patients with chronic kidney disease, including patients on the lower extreme of renal function for which edoxaban use is allowed, will also be determined. The Global ETNA database will also enable comparisons of different treatment practices in patient subgroups by country/region; indication (AF or VTE); gender; patient age, body weight, or body mass index; and others. Furthermore, Global ETNA‐AF and ‐VTE will provide routine care information on edoxaban adherence and how it changes over time, as well as the use of concomitant drugs and dosing of edoxaban in relation to the type and severity of clinical events.

The Global ETNA program is comprehensive and unique in its potential to provide detailed information pertaining to stroke, SEE, VTE, and bleeding, including subtypes and outcomes. Unlike GARFIELD‐AF, GARFIELD‐VTE, GLORIA‐AF, and EORP‐AF, which enrolled patients on various antithrombotic regimens and drugs, edoxaban will be the only oral anticoagulant administered in ETNA.^17^, ^18^, ^19^, ^21^ In addition to addressing questions that randomized clinical trials cannot answer, the integrated analyses of the Global ETNA noninterventional study data will provide extensive insight into edoxaban use in routine clinical care. Furthermore, it provides potential opportunity to analyze the ETNA data in the context of other applicable clinical data sources.

The limitations of noninterventional observation studies will be factored into the interpretation of the results. As is typical of prospective noninterventional studies, the lack of randomization may introduce selection bias, there is no control arm, treatment is not standardized between study sites, and patients may be treated with study drug for varying lengths of time. Additionally, in the ENTA program, clinical events are based on physician diagnosis and assessment (there is light adjudication for ETNA‐AF Europe and ETNA‐VTE Europe).

This program was designed ultimately to provide a global overview of the experience of edoxaban use in its approved indications in routine clinical care, while simultaneously providing sufficient specificity to reflect regional differences. This can potentially help optimize the care of patients receiving anticoagulation with edoxaban. In addition, the ongoing Edoxaban Management in Diagnostic and Therapeutic Procedures (EMIT)‐AF/VTE registry will provide data on the periprocedural management of edoxaban in routine clinical practice in patients with AF or VTE.^22^

The Global ETNA program will be the largest source of prospective routine clinical care data on edoxaban with the planned treatment of >31 000 patients. The program will provide important information on the safety of edoxaban and further information on its effectiveness in patients with AF and VTE, enhancing medical knowledge on edoxaban use in routine clinical care.

## CONFLICT OF INTEREST

RDC reports being a coauthor for ESC Guidelines on Atrial Fibrillation 2010–2012; steering committee member, National Coordinator for Italy; coauthor of APPRAISE‐2, ARISTOTLE, AVERROES, ENGAGE AF‐TIMI 48, Re‐DUAL PCI; and fees, honoraria, and research funding from Sanofi‐Aventis, Boehringer Ingelheim, Bayer, Pfizer, Bristol‐Myers Squibb, Daiichi Sankyo, Novartis, Merck, and Portola. GA reports consultant or speaker fees from Bayer, BMS‐Pfizer, and Daiichi‐Sankyo. CCW reports consulting fees and honoraria from Bayer, Boehringer Ingelheim, and Daiichi Sankyo. MN reports personal fees from Daiichi Sankyo during the conduct of the study; personal fees from Bayer Yakuhin, Ltd., Pfizer Japan Inc., and Bristol‐Myers Squibb K.K. outside the submitted work. KMC reports consulting fees and honoraria from Bayer, Boehringer Ingelheim, Pfizer and Daiichi Sankyo. YK reports being a coauthor for JCS Guidelines on Atrial Fibrillation 2013; coauthor of ENGAGE AF‐TIMI 48; lecture fees from Daiichi Sankyo, Boehringer Ingelheim, Pfizer, Bayer HealthCare, and Bristol‐Myers Squibb; and grant support through his institution from Daiichi Sankyo and Boehringer Ingelheim. YHK is a member of the advisory committee for the ETNA‐AF study, and received honoraria (lecture fees), consulting fees, and research grants from Daiichi Sankyo. TY reports research funding from Bristol‐Myers Squibb, Bayer HealthCare, Daiichi Sankyo, and Mitsubishi‐Tanabe Pharmaceutical; and remuneration from Daiichi Sankyo, Bayer HealthCare, Pfizer, Bristol‐Myers Squibb, Boehringer Ingelheim, Eisai, Toa Eiyo, Takeda, and Ono Pharmaceutical. AC receives consulting fees from AbbVie, ACI Clinical, Aspen, Bayer, Boehringer‐Ingelheim, Bristol‐Myers Squibb, Boston Scientific, CSL Behring, Daiichi‐Sankyo, GlaxoSmithKline, GLG, Guidepoint Global, Johnson and Johnson, Leo Pharma, Medscape, McKinsey, Navigant, ONO, Pfizer, Portola, Sanofi, Temasek Capital, and TRN. WIC and PK report no conflicts of interest. PL, MU, HR, PR, CC, UT, and JK are employees of Daiichi Sankyo.

## Supporting information


**Table 1.** Participating countries in the ETNA program by region
