# Supplementary material for: The global Edoxaban Treatment in routine cliNical prActice (ETNA) noninterventional study program: rationale and design
Source: Clin Cardiol. 2019 Oct 25;42(12):1147–54. doi: 10.1002/clc.23279 (PMC6906985; doi:10.1002/clc.23279)
Supplement: Supplementary file 1 — Table 1. Participating countries in the ETNA program by region [file CLC-42-1147-s001.docx]

**Supplemental Table 1.** Participating countries in the ETNA program by region

| **Europe** | **East and Southeast Asia** | **Japan** |
| --- | --- | --- |
| Germany, Austria, Switzerland, Belgium, Italy, Spain*, the UK, Ireland, the Netherlands, and Portugal* | South Korea, Taiwan, Hong Kong,* and Thailand* | Japan |

*Not participating in ETNA-VTE

ETNA, Edoxaban Treatment in routine cliNical prActice; VTE, venous thromboembolism.
